# An Integrative Bioinformatics Framework Nominates Candidate Limbal Stem-Cell Exosome Cargo for Keratoconus by Coupling Corneal Transcriptomics, Disease-Gene Evidence and Extracellular-Vesicle Repositories

**DOI:** 10.3390/bioengineering13080853

**Published:** 2026-07-24

**Authors:** Chun-Chieh Chao, Hsieh-Tsung Ethan Shen, Bo-Xiang Benjamin Zhang, Ting-Hsuan Chao, Chien-Yi Tu, Chen-hsin Tsai

**Affiliations:** 1 Graduate Institute of Injury Prevention and Control, College of Public Health, Taipei Medical University, Taipei 11031, Taiwan; 2 Department of Emergency Medicine, Taipei Medical University Hospital, Taipei 110301, Taiwan; 3 Department of Emergency Medicine, School of Medicine, College of Medicine, Taipei Medical University, Taipei 11031, Taiwan; 4 YD Bio Limited, Taipei 115603, Taiwan; 5 Division of Clinical Cariology and Endodontology, Department of Oral Rehabilitation, School of Dentistry, Health Sciences University of Hokkaido, Tobetsu 061-0293, Japan; 6 Ph.D. Program in Medical Neuroscience, College of Medical Science and Technology, Taipei Medical University and National Health Research Institutes, Taipei 11031, Taiwan; 7 Department of Molecular and Cell Biology, College of Letter and Science, University of California, Berkeley, CA 94720, USA; 8 Executive Master Program of Business Administration in Biotechnology, Taipei Medical University, Taipei 11031, Taiwan; 9 Department of Ophthalmology, Taipei Medical University Hospital, Taipei 110301, Taiwan

**Keywords:** keratoconus, limbal stem cells, exosomes, extracellular vesicles, transcriptomics, differential expression, protein–protein interaction network, cargo prioritisation, corneal regeneration, reproducible bioinformatics

## Abstract

Background: Keratoconus is a progressive corneal ectasia characterised by extracellular matrix (ECM) loss and an emerging inflammatory component, for which no disease-modifying molecular therapy exists. Exosomes derived from limbal and mesenchymal stem cells are an attractive cell-free therapeutic modality, but the cargo that should be delivered is undefined, and no curated limbal stem-cell (LSC) exosome cargo dataset currently exists. Methods: We reanalysed a public keratoconus corneal RNA-sequencing dataset (GEO: GSE77938; discovery and replication cohorts) with DESeq2, defined a replicated differentially expressed gene (DEG) set, and performed Gene Ontology, KEGG and Reactome enrichment. A high-confidence protein–protein interaction (PPI) network (STRING) identified hub genes. We integrated keratoconus disease-gene evidence (Open Targets Platform) and documented extracellular-vesicle cargo (ExoCarta, Vesiclepedia) and computed a transparent Cargo Prioritization Score (CPS) to nominate candidate LSC-exosome therapeutic cargo. Results: A total of 1677 DEGs were detected in discovery (152 up, 1525 down) and 1380 were replicated. Enrichment was dominated by extracellular matrix organisation; adaptive immune response; and mononuclear cell differentiation. Network analysis nominated ECM and immune hub genes. The CPS prioritised COL1A1, FN1, COL4A1, COL3A1, COL5A1, MMP1 as leading restoration-cargo candidates, all documented as EV cargo and present in the mesenchymal stem-cell EV reference proteome. Conclusions: This fully reproducible, real-data framework provides a ranked, evidence-traceable shortlist of candidate LSC-exosome cargo for keratoconus and an explicit account of current data gaps to guide experimental validation.

## 1. Introduction

Keratoconus is the most common corneal ectasia and a leading indication for corneal transplantation in young adults; it is defined by progressive stromal thinning, biomechanical weakening and disruption of collagen and other ECM components [1,2]. Although historically regarded as a non-inflammatory condition, accumulating tear-film and tissue evidence now supports a low-grade inflammatory and immune contribution to disease progression [3,4,5]. Current management—rigid contact lenses, corneal cross-linking and, ultimately, keratoplasty—addresses biomechanics and optics but does not target the underlying molecular pathology, leaving a clear unmet need for disease-modifying, regenerative approaches.

The corneal epithelium is continuously renewed by limbal epithelial stem cells residing in the limbal niche, whose dysfunction underlies limbal stem-cell deficiency and impaired corneal surface homeostasis [6,7,8]. Stem-cell-derived exosomes—nanoscale extracellular vesicles (EVs) carrying proteins, mRNA and microRNA—have emerged as a cell-free regenerative modality, and limbal-niche- and mesenchymal stem-cell (MSC)-derived EVs accelerate corneal epithelial repair in preclinical models [9,10,11,12,13,14]. A central translational question, however, is which molecular cargo a therapeutic LSC exosome should carry to correct keratoconus pathology. No curated LSC-exosome cargo dataset is available in the major EV repositories, which precludes a purely data-driven cargo definition and instead motivates an integrative, evidence-ranked nomination strategy.

Here we develop a reproducible computational framework that couples real keratoconus corneal transcriptomics with disease-gene evidence, PPI topology and documented EV-cargo competence to prioritise candidate LSC-exosome cargo. We deliberately treat the absence of LSC- and induced-pluripotent-stem-cell (iPSC)–EV cargo data as an explicit, declared limitation rather than imputing it, and we provide a transparent Cargo Prioritization Score whose every component is traceable to a public dataset, public database or stated scoring rule.

## 2. Materials and Methods

**Figure 1:**
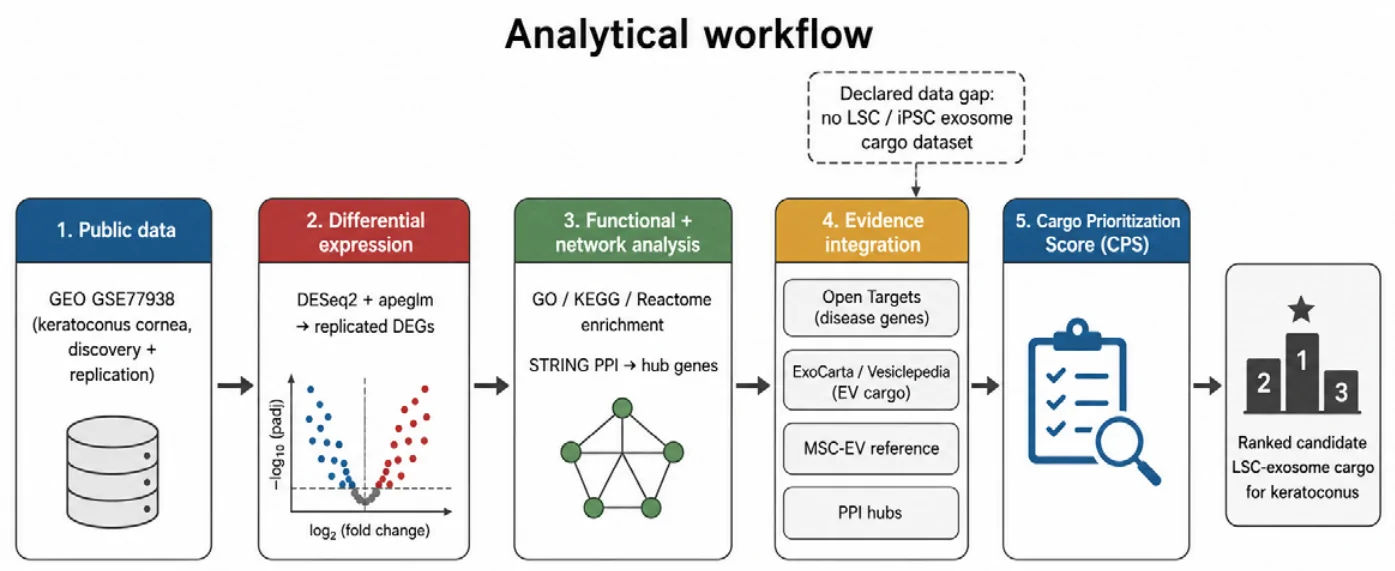
Overall analytical workflow. Public keratoconus corneal transcriptomes (GEO GSE77938) undergo DESeq2 differential expression and replication, followed by GO/KEGG/Reactome enrichment and STRING PPI/hub analysis; disease-gene (Open Targets) and extracellular-vesicle cargo (ExoCarta, Vesiclepedia, MSC-EV reference) evidence are integrated into a transparent Cargo Prioritization Score that ranks candidate LSC-exosome cargo. The absence of an LSC/iPSC exosome cargo dataset is shown as a declared data gap.

The overall analytical workflow, from public-data acquisition through differential expression, functional and network analysis, multi-source evidence integration and Cargo Prioritization Scoring, is summarised in Figure 1.

### 2.1. Dataset and Preprocessing

Processed gene-level RNA-sequencing counts for human keratoconus (KTCN) and non-keratoconus (non-KTCN) corneas were obtained from Gene Expression Omnibus accession GSE77938 [15,16], which comprises an independent discovery cohort (8 KTCN versus 8 control) and a replication cohort (17 KTCN versus 17 control), all corneal tissue (50 samples in total; Table 1). Genes with at least 10 counts in a number of samples equal to the smaller group were retained.

### 2.2. Differential Expression

Differential expression was performed independently in each cohort with DESeq2 [17] using the model ~group (KTCN versus control); log2 fold changes were shrunk with apeglm [18]. Ensembl identifiers were mapped to gene symbols and Entrez IDs (org.Hs.eg.db). DEGs were defined as adjusted *p* < 0.05 (Benjamini–Hochberg) and |log2 fold change| > 1. A replicated (consensus) DEG set was defined as DEGs significant in discovery that were concordant in sign and nominally significant (*p* < 0.05) in the replication cohort.

### 2.3. Functional Enrichment

Gene Ontology Biological Process, KEGG and Reactome over-representation analyses of the consensus DEGs were performed with clusterProfiler [19] and ReactomePA [20] against the set of all tested genes as background (Benjamini–Hochberg *p* < 0.05; redundant GO terms were simplified at a semantic-similarity cutoff of 0.7).

### 2.4. Protein–Protein Interaction Network and Hub Genes

The 400 most significant consensus DEGs (by discovery-adjusted *p*) were submitted to the STRING database [21]; only high-confidence interactions (combined score ≥ 700) were retained. The network was analysed in NetworkX, and hub genes were ranked by degree and betweenness centrality (igraph/NetworkX) [22,23].

### 2.5. Database Integration and Cargo Prioritization Score

Keratoconus disease-gene association scores were retrieved from the Open Targets Platform (disease MONDO_0015486) [24]. Documented human EV-cargo competence and a mesenchymal stem-cell (MSC) EV reference proteome were derived from ExoCarta [25] and Vesiclepedia [26]; EV nomenclature follows MISEV2023 [27]. For each replicated DEG, five components were scaled to [0, 1] or set as indicators: differential-expression evidence (S_DE, scaled |log2 fold change|), disease association (S_disease, Open Targets score), PPI hub centrality (S_hub, scaled degree), documented EV-cargo competence (S_EVcargo) and MSC-EV cargo membership (S_MSC). To integrate these complementary evidence sources into a single ranking metric, we empirically assigned weights that emphasised transcriptomic dysregulation and disease relevance while retaining contributions from network topology and extracellular-vesicle evidence. The resulting Cargo Prioritization Score (CPS) was calculated as: CPS = 0.30·S_DE + 0.25·S_disease + 0.20·S_hub + 0.15·S_EVcargo + 0.10·S_MSC. A disease-gene-cargo-pathway network was assembled for the top-ranked candidates.

## 3. Results

### 3.1. Differentially Expressed Genes in Keratoconus Cornea

**Figure 2:**
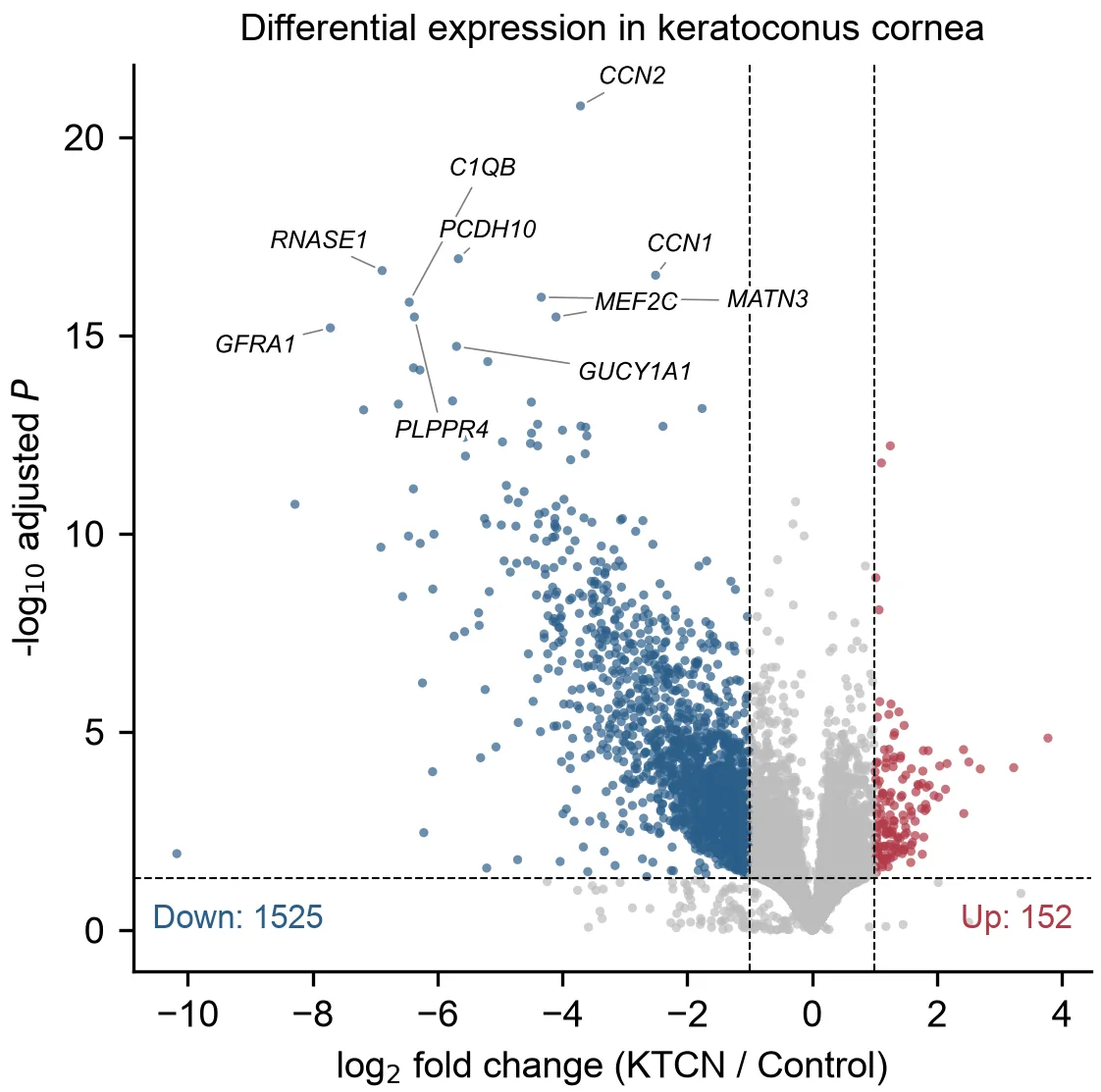
Volcano plot of differential expression in keratoconus versus control cornea (discovery cohort, GSE77938). Coloured points pass adjusted *p* < 0.05 and |log2FC| > 1.

**Figure 3:**
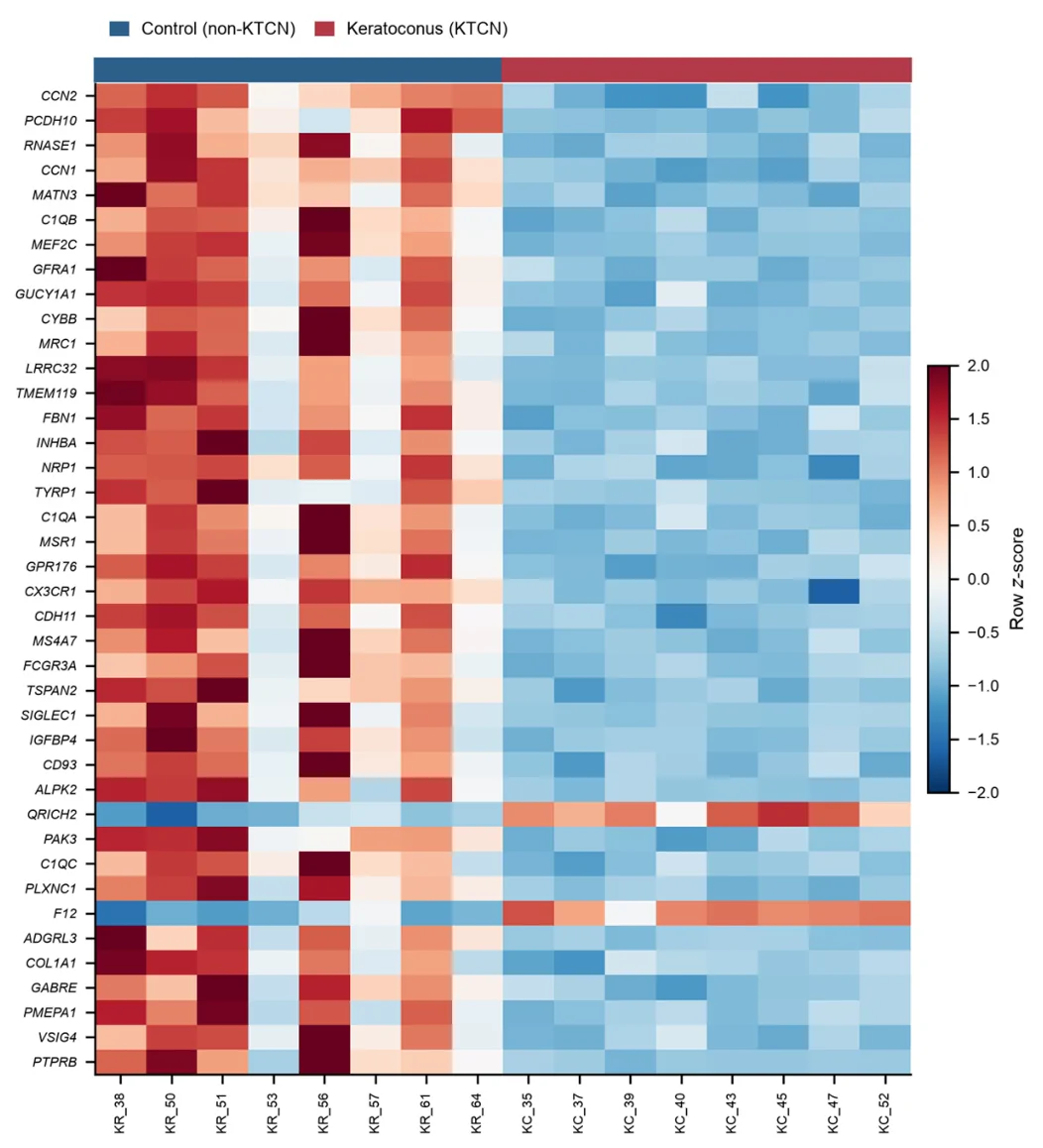
Heatmap of the top 40 replicated DEGs (variance-stabilised expression, row z-score) showing clear separation of keratoconus and control corneas.

Across 27,830 testable genes in the discovery cohort, 1677 genes were differentially expressed (152 up- and 1525 down-regulated in KTCN), a pronounced down-regulation bias consistent with loss of corneal structural transcripts (Figure 2). Of these, 1380 were replicated in the independent cohort and were carried forward as the consensus DEG set (Table 2; Figure 3). The most strongly and consistently dysregulated genes, ranked by discovery adjusted P, are listed in Table 3.

### 3.2. Functional Enrichment

**Figure 4:**
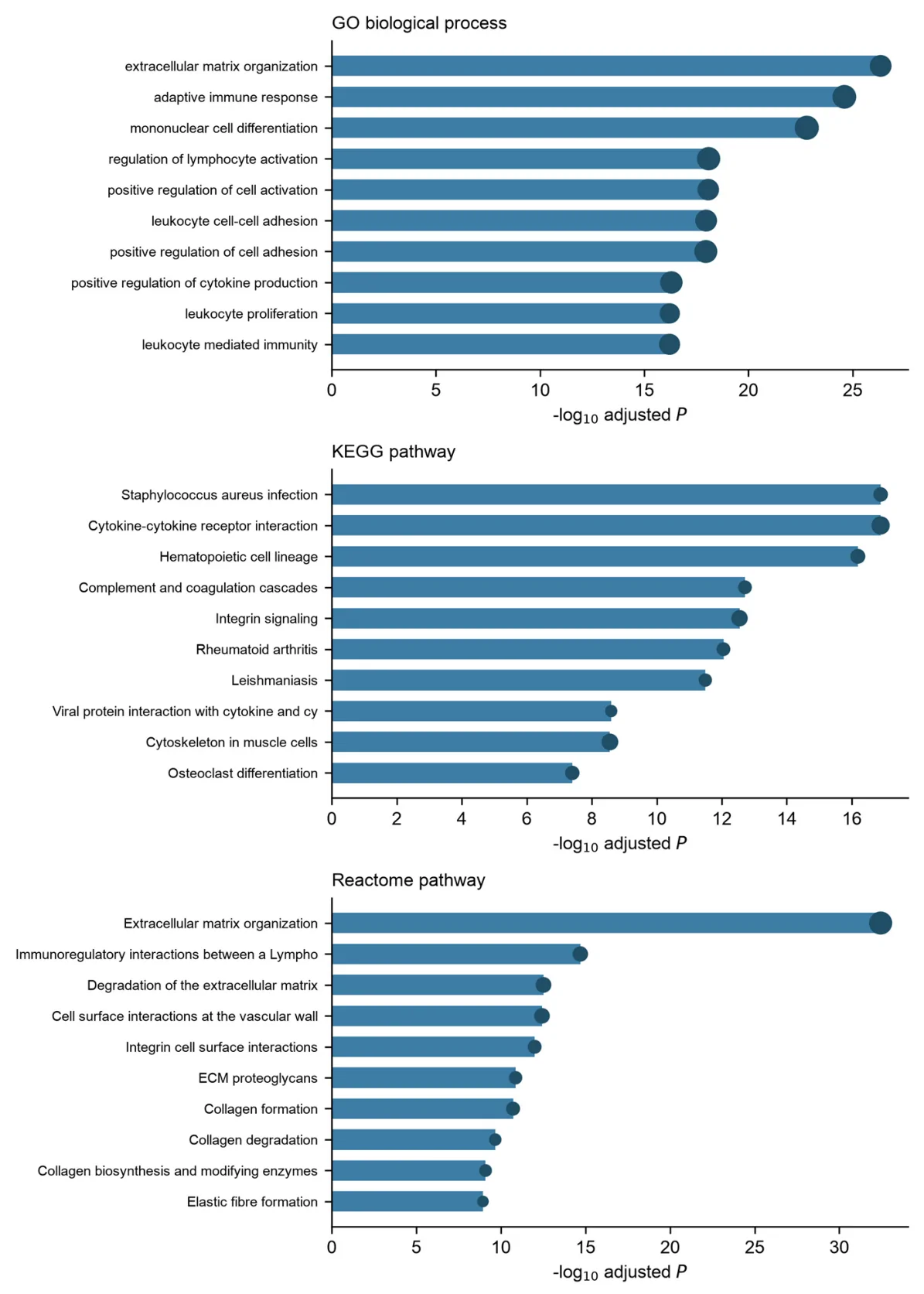
Top enriched GO biological-process, KEGG and Reactome terms for the replicated DEGs (bar length and marker indicate -log10 adjusted *p* and gene count).

The consensus DEGs were strongly enriched for ECM organisation and degradation, collagen and integrin biology, and immune/cytokine signalling across GO, KEGG and Reactome (Figure 4; Table 4). The leading GO term was “extracellular matrix organisation” (adjusted *p* = 4.5 × 10^−27^, 85 genes), and Reactome was led by “extracellular matrix organisation” (adjusted *p* = 3.6 × 10^−33^). This dual ECM-and-immune signature is concordant with the contemporary view of keratoconus pathology [2,5].

Biologically, the dual extracellular-matrix (ECM) and immune signature recapitulate the two convergent axes of keratoconus pathology. The pronounced down-regulation of ECM-organisation, collagen-biosynthesis and integrin programmes is concordant with the progressive stromal thinning, disorganised collagen lamellae and reduced biomechanical strength that define the disease [1,2], whereas the enriched cytokine, complement and leukocyte-activation terms align with the growing recognition of a low-grade inflammatory and immune contribution to keratoconus progression [3,4,5]. The enrichment therefore points not to a single pathway but to a coordinated loss of matrix-maintenance programmes accompanied by immune activation—the biological context in which any candidate therapeutic cargo must be interpreted.

### 3.3. PPI Network and Hub Genes

**Figure 5:**
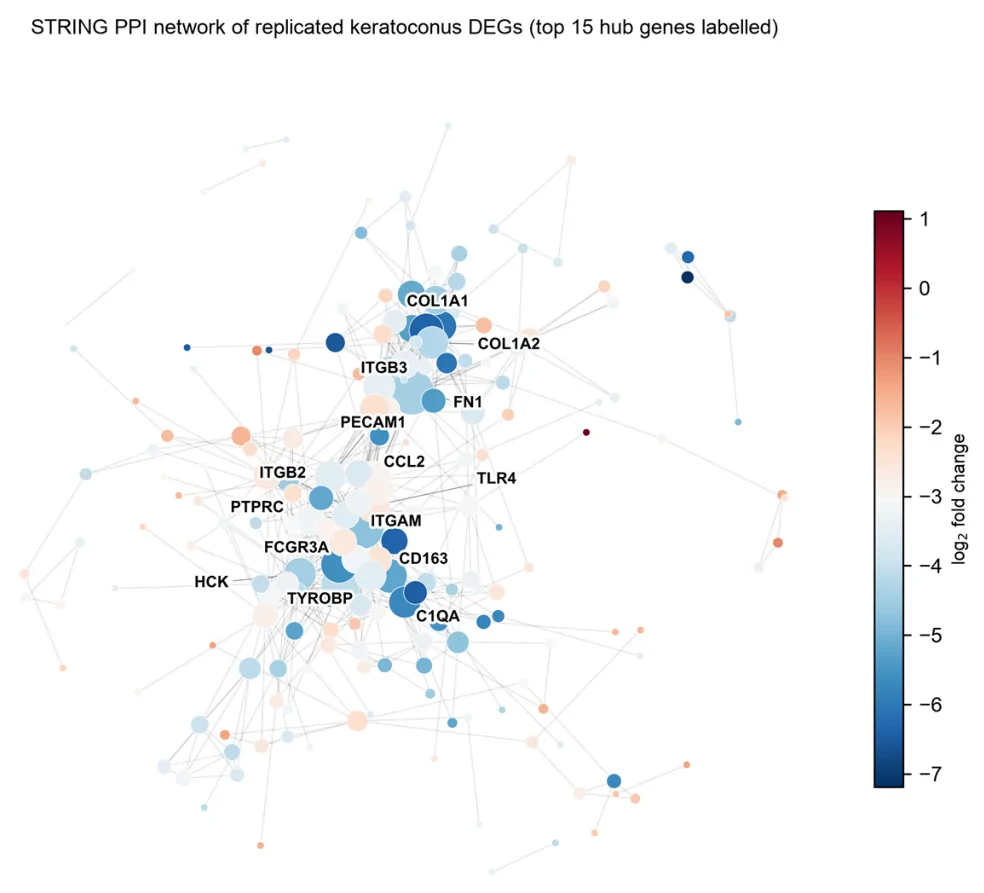
STRING protein–protein interaction network of replicated keratoconus DEGs (combined score ≥ 700). Node size, degree centrality; colour, log2 fold change. Top hub genes are labelled.

A high-confidence STRING network of the consensus DEGs contained 200 connected proteins and 621 edges (Figure 5). The highest-degree hubs—FN1, ITGAM, PTPRC, ITGB2, TYROBP, FCGR3A, CCL2, CD163—comprised both ECM/collagen proteins and immune effectors, all down-regulated in keratoconus (Table 5), pinpointing co-ordinately suppressed modules as rational intervention nodes.

The topology of the network is itself informative. Fibronectin-1 (FN1) and the fibrillar collagens COL1A1/COL1A2 occupy central, high-degree positions, consistent with their role as organising scaffolds of the corneal stroma whose coordinated down-regulation would be expected to destabilise the wider ECM module [1,2]. In parallel, a second hub community of immune effectors (PTPRC/CD45, ITGAM, ITGB2, TYROBP, FCGR3A, CD163 and complement C1QA) reflects the inflammatory axis noted above [3,4]. Because hubs are network-central rather than merely differentially expressed, their coordinated suppression identifies matrix-maintenance and immune-regulatory modules—rather than isolated genes—as the processes a therapeutic exosome would need to influence.

### 3.4. Cargo Prioritisation

**Figure 6:**
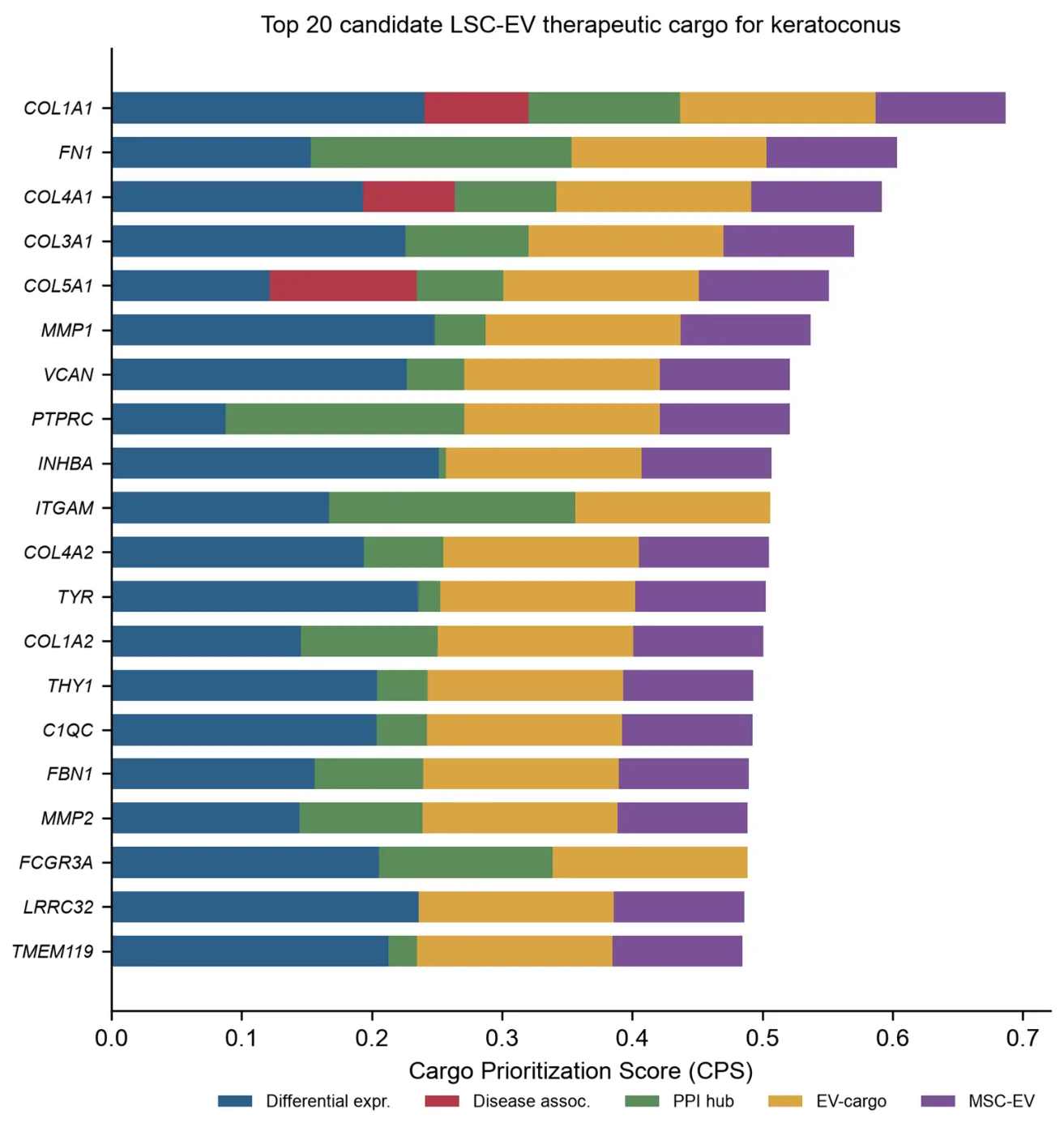
Cargo Prioritization Score (CPS) of the top 20 candidate LSC-exosome cargo, with the contribution of each weighted evidence component shown as stacked segments.

Integrating the five evidence layers, the Cargo Prioritization Score ranked 1253 replicated DEGs. A total of 499 keratoconus-associated targets from Open Targets contributed disease-gene weight, including established keratoconus genes among the top candidates. The highest-scoring candidate cargo were COL1A1, FN1, COL4A1, COL3A1, COL5A1, MMP1, VCAN, PTPRC, INHBA, ITGAM (Figure 6; Table 6)—dominated by ECM and collagen constituents that are down-regulated in keratoconus, documented as EV cargo, and present in the MSC-EV reference proteome, and therefore represent biologically coherent restoration cargo for a limbal stem-cell exosome. Because keratoconus is characterised by ECM loss, these candidates are framed as restoration (replacement) cargo, whereas up-regulated immune candidates would instead represent suppression targets requiring a different cargo modality.

The leading candidates are biologically coherent as putative restoration cargo. COL1A1, the top-ranked gene, together with FN1, COL4A1, COL3A1 and COL5A1, encodes the principal fibrillar and basement-membrane constituents of the corneal stroma whose loss underlies keratoconic thinning; several (COL5A1, COL4A1) additionally carry independent keratoconus disease-gene evidence in Open Targets [24], and all are documented extracellular-vesicle cargo present in the mesenchymal stem-cell EV reference proteome. Their prioritisation is thus driven by convergent, orthogonal evidence rather than by differential expression alone. We emphasise, however, that this ranking nominates hypotheses for experimental testing rather than validated therapeutic targets (Section 4).

### 3.5. Robustness of the Cargo Prioritization Score

Because the CPS weights are assigned a priori rather than learned from outcome data, we tested whether the resulting ranking is robust to the choice of weights (Figure S1, Table 7). The score was recomputed under (i) equal weights, (ii) 10,000 random weight vectors drawn uniformly from the five-component simplex, and (iii) 10,000 ± 50% perturbations of the a priori weights, and each ranking was compared with the original by Spearman rank correlation. The ranking was highly stable: the median Spearman ρ was 0.99 (interquartile range 0.99–1.00) under ±50% perturbation, 0.98 (0.96–0.98) under fully random weights and 0.98 under equal weights, with a median of 80% of the top-20 candidates retained. The leading candidates were essentially invariant: COL1A1 remained the top-ranked gene in every one of the 10,000 perturbed weightings, and FN1, COL4A1 and COL3A1 remained within the top six (median ranks 2, 3 and 4). In particular, COL4A1 and COL3A1—whose adjacency was highlighted during review—retained their relative order in the large majority of schemes (median rank 3, 5th–95th percentile 2–5, versus median rank 4, 3–6). The prioritisation is therefore governed by the convergence of the underlying evidence layers rather than by the precise weight values.

## 4. Discussion

Using only real, publicly available data, this study converts a fragmented evidence landscape into a single, ranked and fully traceable shortlist of candidate limbal stem-cell exosome cargo for keratoconus. The convergence of the differential-expression, network and disease-gene layers on extracellular-matrix and collagen biology is reassuring: it recapitulates the established molecular hallmark of keratoconus and indicates that the prioritisation is anchored in disease-relevant biology rather than analytical artefact.

**Figure 7:**
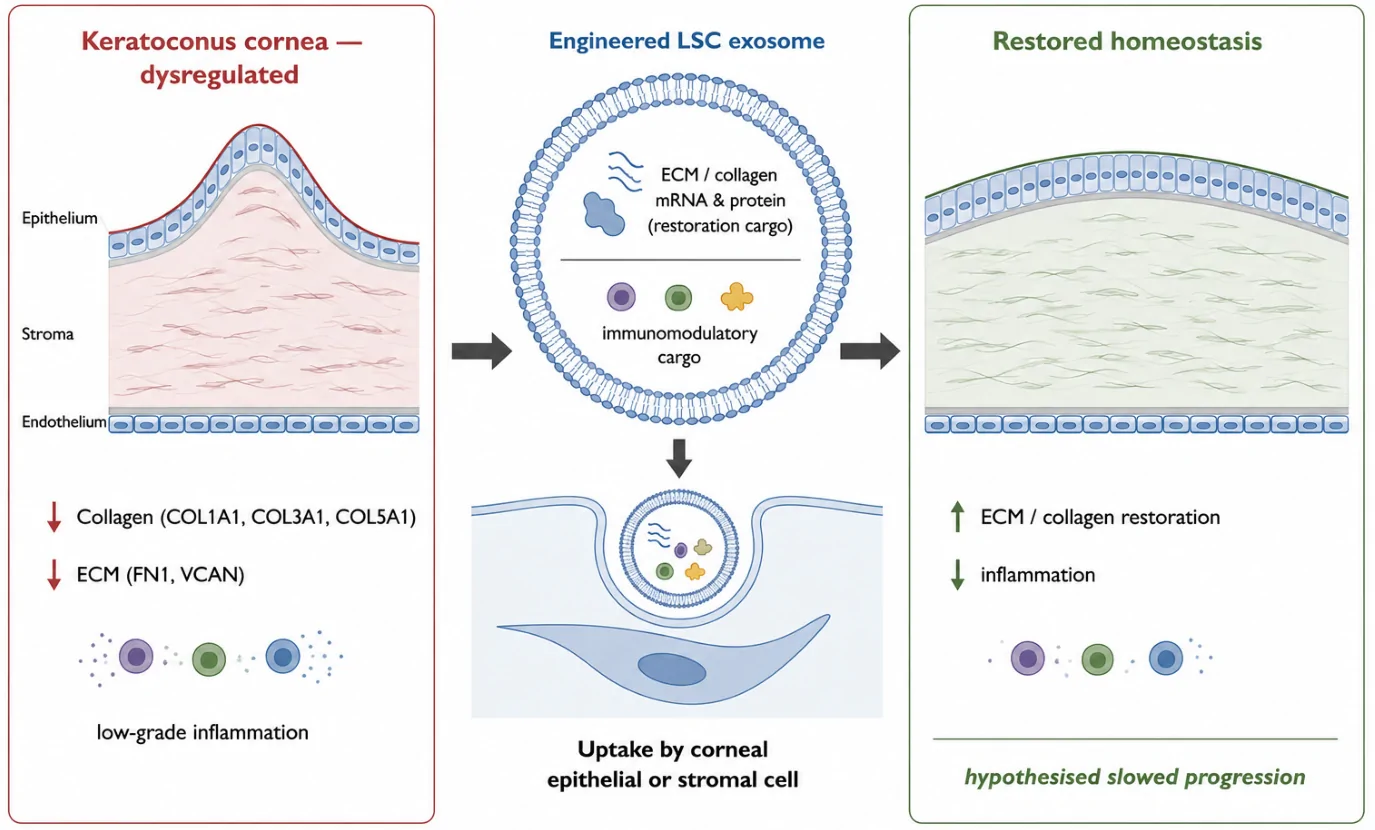
Proposed mechanism-of-action model (conceptual hypothesis). An engineered limbal stem-cell exosome delivers restoration cargo (ECM/collagen mRNA and protein) and immunomodulatory cargo to the keratoconus cornea, hypothesised to restore extracellular-matrix homeostasis and attenuate low-grade inflammation. This model is hypothetical and was not experimentally validated in the present study. This schematic is conceptual and hypothetical; no in vivo, in vitro or other experimental work was performed in this study.

The framework reframes a data gap as a hypothesis-generating opportunity. Because no LSC-exosome cargo repository exists, we do not claim to describe the LSC-EV proteome; instead, we ask which disease-relevant molecules a therapeutic LSC exosome would ideally carry, and we restrict candidates to those for which EV delivery is at least plausible (documented EV cargo) and precedented in a stem-cell EV context (MSC-EV reference). The mechanistic hypothesis—that restoring ECM and collagen cargo, while modulating the immune component, could slow keratoconus progression—is consistent with preclinical evidence that limbal-niche- and MSC-derived EVs promote corneal epithelial and stromal repair, and is depicted in Figure 7.

Two assumptions underlying the framework warrant explicit, critical discussion. First, we treat genes down-regulated in keratoconus as candidate restoration cargo; this is a working hypothesis, not an established principle. Down-regulation marks a molecule as depleted in the diseased tissue, but it does not by itself establish that exogenous re-supply—still less re-supply as exosomal cargo—will restore corneal homeostasis. Second, the presence of a protein in extracellular-vesicle repositories indicates only that it can be EV-associated; it does not demonstrate that engineered limbal stem-cell exosomes can package it in functional form, deliver it to the relevant corneal cells and elicit a matrix-restorative response. The supporting evidence is indirect: preclinical studies show that limbal-niche- and MSC-derived EVs can promote corneal epithelial and stromal repair and modulate inflammation [9,10,11,12,13], but these effects are generally attributable to the composite EV cargo rather than to delivery of any single prioritised protein. The CPS should therefore be read as a hypothesis-ranking device that narrows a large candidate space to a testable shortlist, not as evidence of therapeutic efficacy.

A further caveat concerns the binary annotation of candidates as restoration or suppression cargo. Several hubs are functionally pleiotropic; fibronectin-1 (FN1), for example, is simultaneously a core ECM scaffold and an active modulator of immune-cell adhesion and inflammatory signalling, so its down-regulation cannot be assigned unambiguously to a single therapeutic ‘arm’. The restoration/suppression labels are thus heuristic, and pleiotropic molecules such as FN1 may exert context-dependent, bidirectional effects if delivered in a limbal stem-cell EV; their net role would need to be resolved experimentally rather than inferred from the direction of expression change alone.

The CPS is intentionally simple and transparent: all weights are fixed a priori, every component is traceable to a named public source, and the score can be recomputed or reweighted by any user. This favours interpretability and auditability over black-box optimisation, which we regard as appropriate for a hypothesis-prioritisation tool intended to guide, not replace, experimental validation.

The CPS also occupies a specific niche among computational target-prioritisation strategies. Single-source approaches—such as genetics-anchored disease-gene association scoring (e.g., the Open Targets association score [24]) or network-only guilt-by-association from protein-interaction graphs [21]—each capture a single axis of evidence, whereas the CPS integrates five orthogonal layers (differential expression, disease-gene association, network centrality, EV-cargo competence and stem-cell-EV precedent) into one transparent, reweightable score. Relative to machine-learned prioritisation, its fixed, interpretable weights trade predictive optimisation for auditability, which we regard as appropriate given the absence of ground-truth LSC-EV cargo outcomes on which such a model could be trained. To our knowledge, comparable EV-cargo-aware prioritisation frameworks for ocular-surface disease are not yet established, and the present score is therefore offered as a reproducible starting point rather than a definitive method.

**Figure 8:**
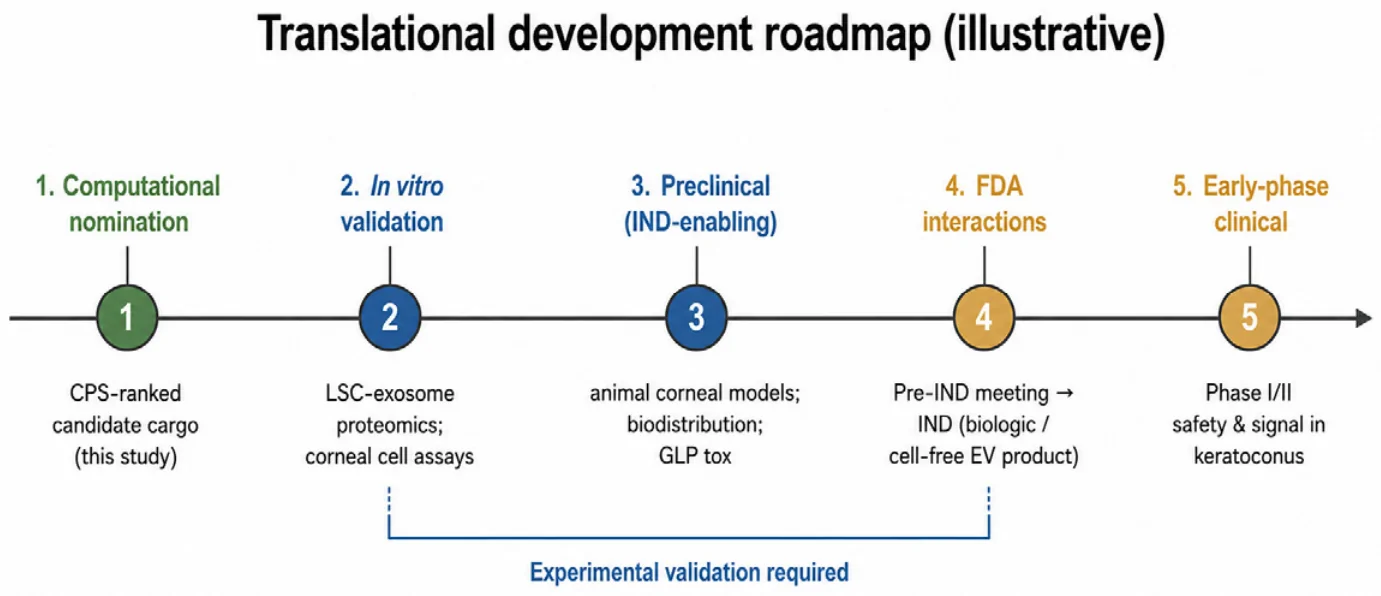
Translational development roadmap (illustrative). Stage-gate path from the present computational cargo nomination through in vitro and IND-enabling preclinical validation, FDA interactions (Pre-IND/IND for a cell-free extracellular-vesicle product) and early-phase clinical evaluation in keratoconus. Stages downstream of nomination require experimental validation. This schematic is conceptual and hypothetical; no in vivo, in vitro or other experimental work was performed in this study.

**Figure 9:**
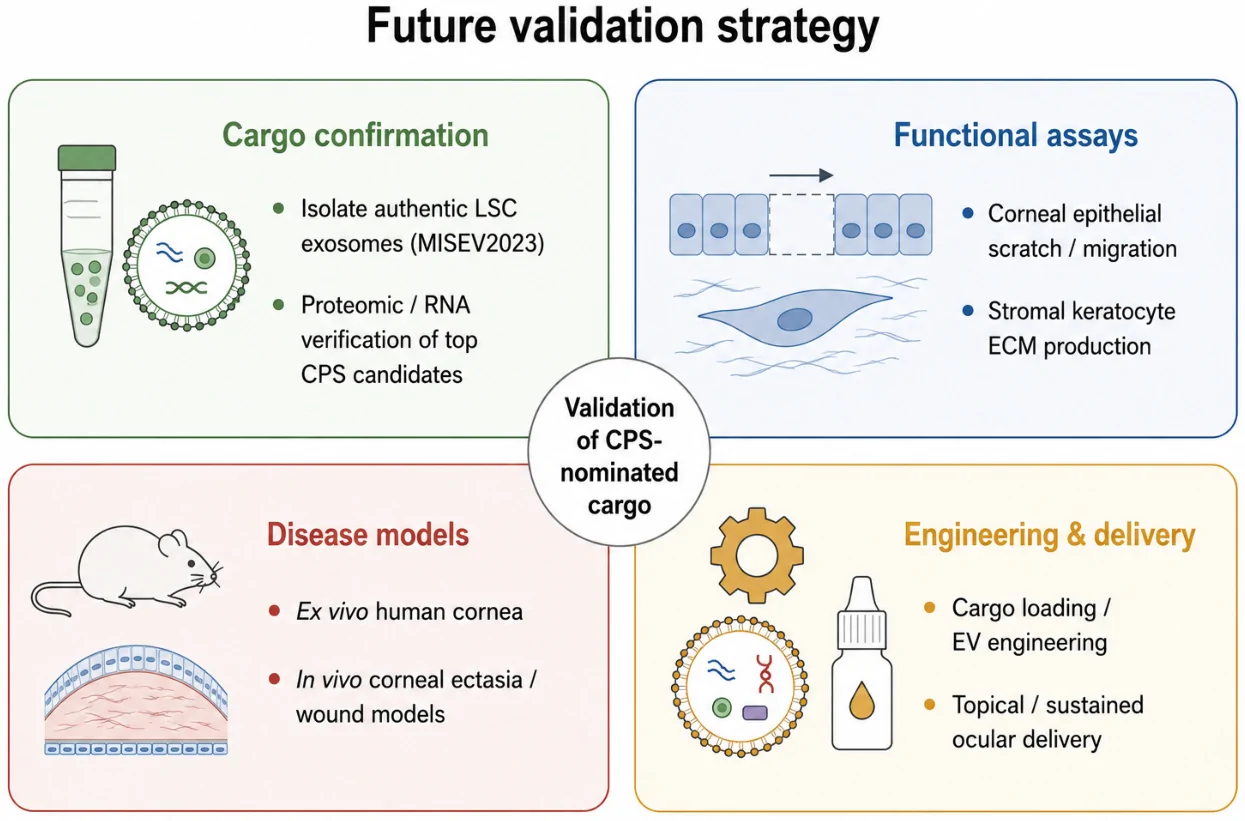
Future validation strategy. Four complementary workstreams—cargo confirmation in authentic LSC exosomes (MISEV2023-compliant), functional corneal cell assays, ex vivo/in vivo disease models, and EV engineering and ocular delivery—required to validate the CPS-nominated candidate cargo. This schematic is conceptual and hypothetical; no in vivo, in vitro or other experimental work was performed in this study.

Finally, we stress that the mechanism-of-action model (Figure 7) and the translational roadmap (Figure 8 and Figure 9) are explicitly hypothetical. Because the study is entirely computational, these schematics depict a proposed, testable path—not an anticipated or likely outcome—and every downstream stage, from cargo confirmation in authentic LSC exosomes to functional and in vivo validation, remains to be performed.

### 4.1. Limitations

Several limitations must be stated explicitly. First, no curated LSC-exosome (or iPSC-exosome) cargo dataset exists in ExoCarta, Vesiclepedia or EVpedia (zero limbal/corneal EV experiments were found); consequently, the LSC-EV cargo is nominated, not measured, and the MSC-EV proteome is used only as the closest available stem-cell EV reference. Second, the transcriptomic evidence derives from a single keratoconus dataset (GSE77938); although it includes an internal replication cohort, external validation in additional cohorts and ocular-surface diseases is required. Third, the public EV repositories are biassed towards well-studied, highly abundant proteins, so EV-cargo competence is a permissive rather than a stringent filter. Fourth, gene-symbol harmonisation between transcriptomic and EV-repository nomenclature is imperfect and may under-count matches. Fifth, the analysis is entirely computational: the prioritised cargo are hypotheses that require proteomic confirmation in authentic LSC exosomes and functional validation in corneal models. Finally, the CPS weights, while transparent, are expert-chosen and not learned from outcome data.

Several further limitations follow. The transcriptomic evidence derives from a single keratoconus study (GSE77938); although it comprises internal discovery and replication cohorts, these are not mutually independent, the combined sample size is modest (n = 50), and no fully independent external cohort was available, so the DEG set and the resulting prioritisation require confirmation in additional, independent keratoconus datasets. Moreover, because no LSC-EV cargo repository exists, we used the MSC-EV proteome as the closest available stem-cell-EV reference; however, limbal stem cells are of epithelial origin whereas mesenchymal stem cells are stromal/mesenchymal, and their secreted-vesicle cargo may differ substantially—for instance, in epithelial- versus stromal-associated proteins. The MSC-EV layer therefore provides only an approximate proxy for EV-deliverability, and candidates should ultimately be validated against the cargo of authentic limbal stem-cell exosomes.

### 4.2. Translational Outlook

Beyond target nomination, the candidate cargo identified here define a concrete translational path. The in silico shortlist (Figure 8, stage 1) would first require confirmation that the prioritised molecules are genuinely present in authentic limbal stem-cell exosomes, followed by functional and preclinical validation, regulatory interactions and early-phase clinical evaluation of a cell-free exosome product. A structured validation programme spanning cargo confirmation, functional assays, disease models and EV engineering/delivery is outlined in Figure 9. We emphasise that all stages downstream of the present computational nomination remain to be performed; the framework is intended to focus and de-risk, not to substitute, that experimental work.

## 5. Conclusions

We present a reproducible, integrity-constrained bioinformatics framework that nominates and ranks candidate limbal stem-cell exosome cargo for keratoconus by integrating real corneal transcriptomics, disease-gene evidence, PPI topology and extracellular-vesicle repositories. The approach yields an evidence-traceable shortlist led by ECM/collagen restoration cargo, an explicit map of current data gaps, and a transparent scoring scheme that is directly testable. It provides a rational starting point for designing engineered therapeutic exosomes for corneal ectasia and is readily generalisable to other ocular-surface diseases as suitable datasets become available.

## Figures and Tables

**Table 1:** Keratoconus corneal transcriptomic cohorts analysed (GEO accession GSE77938). KTCN, keratoconus; control, non-keratoconus.

Cohort	KTCN (n)	Control (n)	Total (n)	Tissue	Assay
Discovery	8	8	16	Cornea	RNA-seq
Replication	17	17	34	Cornea	RNA-seq
Total	25	25	50	Cornea	RNA-seq

**Table 2:** Differential-expression summary (GSE77938).

Metric	Value
Genes tested (discovery)	27,830
DEGs (discovery, *p*adj < 0.05, |log2FC| > 1)	1677
up-regulated	152
down-regulated	1525
Genes tested (replication)	23,008
DEGs (replication)	3236
Replicated consensus DEGs	1380

**Table 4:** Representative enriched terms (top five per database).

Source	Term	adj. *p*	Count
GO BP	Extracellular matrix organisation	4.52 × 10^−27^	85
GO BP	Adaptive immune response	2.49 × 10^−25^	102
GO BP	Mononuclear cell differentiation	1.59 × 10^−23^	106
GO BP	Regulation of lymphocyte activation	8.17 × 10^−19^	98
GO BP	Positive regulation of cell activation	8.47 × 10^−19^	82
KEGG	Staphylococcus aureus infection	1.3 × 10^−17^	34
KEGG	Cytokine–cytokine receptor interaction	1.3 × 10^−17^	57
KEGG	Hematopoietic cell lineage	6.5 × 10^−17^	36
KEGG	Complement and coagulation cascades	1.93 × 10^−13^	30
KEGG	Integrin signalling	2.84 × 10^−13^	45
Reactome	Extracellular matrix organisation	3.59 × 10^−33^	96
Reactome	Immunoregulatory interactions between a Lymphoid and a non-Lymphoid cell	2.03 × 10^−15^	39
Reactome	Degradation of the extracellular matrix	3.05 × 10^−13^	41
Reactome	Cell surface interactions at the vascular wall	3.73 × 10^−13^	40
Reactome	Integrin cell surface interactions	1.02 × 10^−12^	31

**Table 5:** Top 12 PPI hub genes by degree centrality.

Gene	Degree	Betweenness	log2FC (Disc)
FN1	36	0.153	−4.43
ITGAM	34	0.0659	−4.74
PTPRC	33	0.0955	−2.96
ITGB2	28	0.0862	−4.28
TYROBP	28	0.0518	−4.09
FCGR3A	24	0.0256	−5.6
CCL2	23	0.126	−2.93
CD163	21	0.0293	−5.24
COL1A1	21	0.018	−6.39
TLR4	20	0.023	−2.51
HCK	19	0.0465	−4.45
COL1A2	19	0.0062	−4.25

**Table 6:** Top 20 CPS-ranked candidate LSC-exosome cargo for keratoconus.

Rank	Gene	Dir.	log2FC	Disease	Degree	EV	MSC-EV	CPS
1	COL1A1	down	−6.39	0.318	21	1	True	0.687
2	FN1	down	−4.43	0	36	1	True	0.603
3	COL4A1	down	−5.33	0.281	14	1	True	0.592
4	COL3A1	down	−6.06	0	17	1	True	0.57
5	COL5A1	down	−3.73	0.451	12	1	True	0.551
6	MMP1	down	−6.56	0	7	1	True	0.537
7	VCAN	down	−6.08	0	8	1	True	0.521
8	PTPRC	down	−2.96	0	33	1	True	0.521
9	INHBA	down	−6.63	0	1	1	True	0.507
10	ITGAM	down	−4.74	0	34	1	False	0.506
11	COL4A2	down	−5.34	0	11	1	True	0.505
12	TYR	down	−6.28	0	3	1	True	0.502
13	COL1A2	down	−4.25	0	19	1	True	0.501
14	THY1	down	−5.57	0	7	1	True	0.493
15	C1QC	down	−5.55	0	7	1	True	0.492
16	FBN1	down	−4.5	0	15	1	True	0.49
17	MMP2	down	−4.23	0	17	1	True	0.489
18	FCGR3A	down	−5.6	0	24	1	False	0.489
19	LRRC32	down	−6.28	0	0	1	True	0.486
20	TMEM119	down	−5.76	0	4	1	True	0.485

**Table 7:** Sensitivity of the CPS ranking to the weighting scheme. Spearman ρ is computed between each alternative ranking and the a priori ranking; top-20 retention is the fraction of the original top-20 candidates that remain in the top 20.

Weighting Scheme	Spearman ρ (Median [IQR])	Minimum ρ	Top-20 Retained (Median)
±50% perturbation (n = 10,000)	0.99 [0.99–1.00]	0.93	80%
Random simplex (n = 10,000)	0.98 [0.96–0.98]	0.59	60%
Equal weights	0.98	—	80%

**Table 3:** Top 15 replicated DEGs ranked by discovery adjusted *p*.

Gene	log2FC (Disc)	*p*adj (Disc)	log2FC (Rep)	*p*adj (Rep)
CCN2	−3.71	1.64 × 10^−21^	−3.27	5.17 × 10^−13^
PCDH10	−5.67	1.17 × 10^−17^	−2.9	1.28 × 10^−5^
RNASE1	−6.89	2.33 × 10^−17^	−6.24	2.37 × 10^−17^
CCN1	−2.51	3.04 × 10^−17^	−2.63	2.98 × 10^−15^
MATN3	−4.34	1.09 × 10^−16^	−3.9	2.24 × 10^−17^
C1QB	−6.45	1.46 × 10^−16^	−5.63	7.33 × 10^−19^
MEF2C	−4.1	3.44 × 10^−16^	−3.24	1.15 × 10^−14^
GFRA1	−7.72	6.49 × 10^−16^	−3.9	5.12 × 10^−8^
GUCY1A1	−5.7	1.9 × 10^−15^	−4.86	1.18 × 10^−24^
CYBB	−5.2	4.59 × 10^−15^	−5.03	3.62 × 10^−14^
MRC1	−6.39	6.6 × 10^−15^	−5.34	1.07 × 10^−15^
LRRC32	−6.28	7.49 × 10^−15^	−4.67	1.11 × 10^−19^
TMEM119	−5.76	4.51 × 10^−14^	−4.34	9.05 × 10^−17^
FBN1	−4.5	4.84 × 10^−14^	−4.21	1.94 × 10^−25^
INHBA	−6.63	5.43 × 10^−14^	−5.24	3.03 × 10^−14^

## Data Availability

All primary data are public: keratoconus transcriptomes from GEO accession GSE77938; disease-gene associations from the Open Targets Platform (MONDO_0015486); EV cargo from ExoCarta and Vesiclepedia; interactions from STRING. All derived tables generated in this study are provided as Supplementary Materials.

## Supplementary Materials

The following supporting information can be downloaded at: https://www.mdpi.com/article/10.3390/bioengineering13080853/s1, Figure S1: (sensitivity.png) a sensitivity analysis; Table S1: (DE_discovery.csv, DE_replication.csv) full differential-expression results; Table S2: (DEGs_consensus.csv) replicated DEG set; Table S3: (enrich_GO_BP.csv, enrich_KEGG.csv, enrich_Reactome.csv) full enrichment results; Table S4: (ppi_edges.csv, ppi_node_centrality.csv, ppi_hub_genes.csv) PPI network and hubs; Table S5: (opentargets_keratoconus.csv) Open Targets associations; Table S6: (cargo_prioritization_CPS.csv) full CPS ranking; Table S7: (disease_gene_cargo_pathway_edges.csv) integrated network edges.

## Abbreviations

The following abbreviations are used in this manuscript:

BH	Benjamini–Hochberg
BP	biological process (gene ontology)
CPS	Cargo Prioritization Score
DE	differential expression
DEG	differentially expressed gene
ECM	extracellular matrix
EV	extracellular vesicle
FDA	Food and Drug Administration
GEO	Gene Expression Omnibus
GLP	Good laboratory practice
GO	Gene ontology
IND	Investigational new drug
iPSC	induced pluripotent stem cell
KEGG	Kyoto Encyclopedia of Genes and Genomes
KTCN	keratoconus
Log2FC	log2 fold change
LSC	limbal stem cell
LSCD	limbal stem-cell deficiency
miRNA	microRNA
MISEV	Minimal Information for Studies of Extracellular Vesicles
mRNA	messenger RNA
MSC	mesenchymal stem cell
PPI	protein–protein interaction
RNA-seq	RNA sequencing
VST	variance-stabilising transformation

## References

[1] Santodomingo-Rubido J., Carracedo G., Suzaki A., Villa-Collar C., Vincent S.J., Wolffsohn J.S. (2022). Keratoconus: An updated review. Contact Lens Anterior Eye J. Br. Contact Lens Assoc..

[2] Hao X.D., Gao H., Xu W.H., Shan C., Liu Y., Zhou Z.X., Wang K., Li P.F. (2022). Systematically Displaying the Pathogenesis of Keratoconus via Multi-Level Related Gene Enrichment-Based Review. Front. Med..

[3] McMonnies C.W. (2015). Inflammation and keratoconus. Optom. Vis. Sci. Off. Publ. Am. Acad. Optom..

[4] Zhang H., Cao X., Liu Y., Wang P., Li X. (2021). Tear Levels of Inflammatory Cytokines in Keratoconus: A Meta-Analysis of Case-Control and Cross-Sectional Studies. BioMed Res. Int..

[5] Galvis V., Sherwin T., Tello A., Merayo J., Barrera R., Acera A. (2015). Keratoconus: An inflammatory disorder?. Eye.

[6] Deng S.X., Borderie V., Chan C.C., Dana R., Figueiredo F.C., Gomes J.A.P., Pellegrini G., Shimmura S., Kruse F.E. (2019). The International Limbal Stem Cell Deficiency Working Group. Global Consensus on Definition, Classification, Diagnosis, and Staging of Limbal Stem Cell Deficiency. Cornea.

[7] Li S., Sun H., Chen L., Fu Y. (2024). Targeting limbal epithelial stem cells: Master conductors of corneal epithelial regeneration from the bench to multilevel theranostics. J. Transl. Med..

[8] Nuzzi A., Pozzo Giuffrida F., Luccarelli S., Nucci P. (2022). Corneal Epithelial Regeneration: Old and New Perspectives. Int. J. Mol. Sci..

[9] Zhou T., Huang X., Tan Y., Shen J., Zhou X., Li G., Wang W. (2026). Limbal niche cell-derived exosomes accelerate corneal epithelial repair through PI3K/Akt activation and FOXO3 inhibition. Exp. Eye Res..

[10] Xu Y., Wei C., Ma L., Zhao L., Li D., Lin Y., Zhou Q., Xie L., Wang F. (2025). 3D mesenchymal stem cell exosome-functionalized hydrogels for corneal wound healing. J. Control. Release Off. J. Control. Release Soc..

[11] Tang Q., Lu B., He J., Chen X., Fu Q., Han H., Luo C., Yin H., Qin Z., Lyu D. (2022). Exosomes-loaded thermosensitive hydrogels for corneal epithelium and stroma regeneration. Biomaterials.

[12] Yu F., Zhao X., Wang Q., Fang P.H., Liu L., Du X., Li W., He D., Zhang T., Bai Y. (2024). Engineered Mesenchymal Stromal Cell Exosomes-Loaded Microneedles Improve Corneal Healing after Chemical Injury. ACS Nano.

[13] Williams T., Salmanian G., Burns M., Maldonado V., Smith E., Porter R.M., Song Y.H., Samsonraj R.M. (2023). Versatility of mesenchymal stem cell-derived extracellular vesicles in tissue repair and regenerative applications. Biochimie.

[14] Massoumi H., Amin S., Soleimani M., Momenaei B., Ashraf M.J., Guaiquil V.H., Hematti P., Rosenblatt M.I., Djalilian A.R., Jalilian E. (2023). Extracellular-Vesicle-Based Therapeutics in Neuro-Ophthalmic Disorders. Int. J. Mol. Sci..

[15] Kabza M., Karolak J.A., Rydzanicz M., Szcześniak M.W., Nowak D.M., Ginter-Matuszewska B., Polakowski P., Ploski R., Szaflik J.P., Gajecka M. (2017). Collagen synthesis disruption and downregulation of core elements of TGF-β, Hippo, and Wnt pathways in keratoconus corneas. Eur. J. Hum. Genet. EJHG.

[16] Davis S., Meltzer P.S. (2007). GEOquery: A bridge between the Gene Expression Omnibus (GEO) and BioConductor. Bioinformatics.

[17] Love M.I., Huber W., Anders S. (2014). Moderated estimation of fold change and dispersion for RNA-seq data with DESeq2. Genome Biol..

[18] Zhu A., Ibrahim J.G., Love M.I. (2019). Heavy-tailed prior distributions for sequence count data: Removing the noise and preserving large differences. Bioinformatics.

[19] Yu G., Wang L.G., Han Y., He Q.Y. (2012). clusterProfiler: An R package for comparing biological themes among gene clusters. Omics A J. Integr. Biol..

[20] Yu G., He Q.Y. (2016). ReactomePA: An R/Bioconductor package for reactome pathway analysis and visualization. Mol. Biosyst..

[21] Szklarczyk D., Kirsch R., Koutrouli M., Nastou K., Mehryary F., Hachilif R., Gable A.L., Fang T., Doncheva N.T., Pyysalo S. (2023). The STRING database in 2023: Protein-protein association networks and functional enrichment analyses for any sequenced genome of interest. Nucleic Acids Res..

[22] Csárdi G., Nepusz T. (2006). The Igraph Software Package for Complex Network Research. https://api.semanticscholar.org/CorpusID:16923281.

[23] Hagberg A.A., Schult D.A., Swart P.J., Hagberg J. (2008). Exploring Network Structure, Dynamics, and Function Using NetworkX. Proceedings of the Python in Science Conference. https://api.semanticscholar.org/CorpusID:16050699.

[24] Ochoa D., Hercules A., Carmona M., Suveges D., Baker J., Malangone C., Lopez I., Miranda A., Cruz-Castillo C., Fumis L. (2023). The next-generation Open Targets Platform: Reimagined, redesigned, rebuilt. Nucleic Acids Res..

[25] Keerthikumar S., Chisanga D., Ariyaratne D., Al Saffar H., Anand S., Zhao K., Samuel M., Pathan M., Jois M., Chilamkurti N. (2016). ExoCarta: A Web-Based Compendium of Exosomal Cargo. J. Mol. Biol..

[26] Pathan M., Fonseka P., Chitti S.V., Kang T., Sanwlani R., Van Deun J., Hendrix A., Mathivanan S. (2019). Vesiclepedia 2019: A compendium of RNA, proteins, lipids and metabolites in extracellular vesicles. Nucleic Acids Res..

[27] Welsh J.A., Goberdhan D.C.I., O’Driscoll L., Buzas E.I., Blenkiron C., Bussolati B., Cai H., Di Vizio D., Driedonks T.A.P., Erdbrügger U. (2024). Minimal information for studies of extracellular vesicles (MISEV2023): From basic to advanced approaches. J. Extracell. Vesicles.

